# Bank-speed alignment: a conceptual framework for understanding indoor track performance

**DOI:** 10.3389/fspor.2026.1845340

**Published:** 2026-06-03

**Authors:** Kyle Barnes

**Affiliations:** Movement Science Department, Grand Valley State University, Allendale, MI, United States

**Keywords:** bank angle, competitive fairness, curve running biomechanics, indoor track performance, pacing technology, running economy, running velocity, track geometry

## Abstract

Indoor track performance is shaped by interactions among athlete characteristics, technology, and the structural environment of competition, yet the role of track geometry is not fully integrated into existing performance frameworks. While traditional approaches emphasize physiological and biomechanical factors internal to the athlete, features such as turn radius and bank angle may influence how running velocity is produced and maintained during curved running. The purpose of this conceptual analysis was to develop a framework for interpreting how track geometry interacts with running velocity to influence the mechanical demands of indoor track performance. Drawing on established biomechanical principles, including centripetal force and banked-surface dynamics, this work introduces the concept of bank-speed alignment, defined as the relationship between running velocity, turn radius, and bank angle under simplified mechanical conditions. This framework is presented as a conceptual synthesis of existing mechanical relationships rather than a new derivation or validated model of performance. The analysis illustrates that the bank angle associated with the mechanical reference condition is inherently velocity-dependent and therefore cannot be uniformly satisfied across sprint, middle-, and long-distance events. As a result, contemporary indoor track designs—typically constrained to bank angles of approximately 10°–12°—may differ in how closely they align with the velocity demands of different events, which may be associated with differences in mechanical demands during curved running. These relationships are intended to provide a basis for interpreting variability in performance conditions across facilities and events, while situating track geometry within a broader system that includes technological and contextual factors. Overall, this framework is intended as a hypothesis-generating perspective that reframes indoor track performance as an emergent outcome of interactions among athlete capacity, environmental structure, and competition context.

## Introduction

Over the past two decades, the field of sports science has undergone rapid evolution, driven by advances in technology, biomechanics, physiology, and performance analytics. These developments have enabled increasingly sophisticated approaches to athlete monitoring, training prescription, and performance optimization. Wearable technologies, high-resolution motion capture systems, and integrated data platforms now allow practitioners to quantify training loads, biomechanical patterns, and physiological responses with unprecedented precision (1, 2).

Within track and field, performance across running events—from sprints through middle- and long-distance races—has traditionally been interpreted through physiological and biomechanical constructs such as maximal oxygen uptake (VO_2_max), lactate threshold, force production, and running economy (3). While these models have provided a strong foundation for understanding performance, they primarily emphasize characteristics internal to the athlete. Increasingly, however, performance is recognized as an outcome shaped not only by athlete capacity, but also by the environment in which performance occurs. This perspective is particularly relevant to track running, where the physical structure of the competition environment directly constrains how movement is produced and sustained.

Among these environmental factors, the geometry of the track represents one of the most fundamental yet comparatively underexamined determinants of performance. This limitation is especially evident in indoor track, where facility design varies substantially across venues. Unlike outdoor tracks, which are globally standardized at 400 m, indoor tracks differ in key geometric characteristics including lane distance, turn radius, and bank angle (4, 5). These structural differences create distinct mechanical environments that influence how athletes generate force, negotiate curves, and maintain velocity.

The influence of track geometry is most pronounced in events that involve sustained or repeated curve running, including the 200 m, 400 m, and all middle- and long-distance races. In these events, athletes must continuously generate centripetal force to maintain their trajectory, with the magnitude of this requirement determined by both running velocity and track radius. Banking modifies how this force is produced and distributed, altering the mechanical demands placed on the athlete and potentially influencing how velocity is maintained during curved running.

Despite these effects, indoor track environments are often treated as functionally equivalent. Athletes and coaches frequently describe tracks as “fast” or “slow,” yet these descriptors lack a clear mechanistic basis. Existing explanations often emphasize surface properties, competition conditions, or pacing strategies, but do not fully account for the role of track geometry in shaping performance conditions. This disconnect reflects a broader gap in sports science: the limited integration of structural environmental constraints into models of athletic performance.

Prior work has examined aspects of curve running mechanics and track design, including experimental investigations of bend running (6), biomechanical modeling of curved running dynamics (7), and optimal control approaches linking running strategy and track geometry (8). These approaches demonstrate that track geometry influences performance through its interaction with force production, curvature, and pacing strategy. However, existing work has largely focused on specific modeling approaches or event contexts rather than providing an integrated framework connecting these relationships across events and performance environments.

The purpose of this conceptual analysis is to develop a framework that explains indoor track performance through the interaction between running velocity and track geometry. Central to this framework is the concept of bank-speed alignment, which describes how the relationship between velocity, turn radius, and bank angle influences mechanical conditions during curved running. Importantly, the present framework does not introduce a new mechanical law or validated performance model. Rather, it synthesizes established principles of banked-turn mechanics and applies them as an interpretive heuristic for understanding how indoor track geometry may interact with running velocity across events. In this sense, the contribution of this work is conceptual and integrative, linking existing biomechanical relationships across performance contexts rather than deriving new ones. By situating indoor track configuration as a meaningful determinant of performance conditions—rather than a passive background condition—this work aims to provide a more mechanistic and applied understanding of performance across indoor track events from the 200 m through distance races.

## Performance as an interaction between athlete, technology and environment

Contemporary models of athletic performance conceptualize performance as the result of interacting systems rather than isolated physiological variables (9, 10). Within this framework, athlete characteristics, environmental constraints, and task demands jointly shape movement coordination and performance outcomes. In track running, this perspective provides a theoretical basis for understanding how features of the competition environment—such as track geometry—may influence how speed is produced and sustained across sprint, middle-, and long-distance events.

Within this systems-based framework, running performance reflects the integration of multiple interacting determinants, including mechanical, neuromuscular, and metabolic factors that collectively influence how velocity is produced and sustained. Constructs such as running economy—often defined as the energetic cost of running at a given velocity—represent one component of this broader system and are particularly relevant in middle- and long-distance events (11). However, the relative contribution of these factors varies across event types. Sprint performance is more strongly influenced by force–time characteristics, ground contact dynamics, and bend-specific mechanics, whereas middle- and long-distance performance is more closely associated with mechanical efficiency and energetic cost. Importantly, these determinants are not governed solely by internal physiology. External conditions—including footwear, surface properties, and the structural characteristics of the running environment—shape the conditions under which force is generated and applied, rather than independently determining performance outcomes (12).

These external influences are evident in recent technological developments in track and field. Carbon fiber–plated footwear, for example, has been shown to influence running economy under certain conditions by altering the mechanical interaction between the athlete and the ground (13, 14). Similarly, pacing light systems provide external feedback that may influence pacing behavior during competition (15, 16). In the context of the present manuscript, these factors are treated as contextual modifiers that operate within, rather than replace, the structural constraints imposed by track geometry.

While technological innovations may influence performance, their effects occur within the constraints imposed by the physical environment. In indoor track, this environment is highly structured but not uniform. Unlike outdoor tracks, indoor tracks vary in key geometric characteristics, including track size (lane distance), turn radius, and bank angle, all of which directly influence the mechanical demands of running—particularly during curved sections.

From this perspective, performance may be conceptualized as arising from interactions among three primary components: the athlete, the technological environment, and the structural environment. The athlete contributes physiological capacity, neuromuscular coordination, and movement strategy. Technology may modify energy return and pacing behavior but does not eliminate the constraints imposed by track geometry. The structural environment—defined by the configuration of the track—acts as a constraint that shapes how forces must be generated and applied to maintain velocity and trajectory. This perspective is consistent with modeling approaches demonstrating that environmental constraints such as curvature and track geometry influence force production and movement strategy during running (8).

The geometry of the track imposes consistent mechanical demands within a given facility, yet these demands vary systematically across facilities. In particular, turn radius and bank angle shape how athletes generate and apply force while navigating curves, defining the mechanical conditions under which movement is produced rather than directly determining performance outcomes.

These components interact dynamically rather than independently. Changes in athlete capability or technological inputs may alter how athletes engage with environmental constraints but do not remove those constraints. As a result, performance reflects not only athlete capacity, but also the degree to which environmental demands align with the velocity requirements of the event.

This interaction is particularly relevant in indoor track, where the relationship between velocity, radius, and bank angle governs the mechanical demands of curved running. Because these demands vary across events and facilities, performance cannot be fully understood without considering how athletes interact with track geometry under specific competitive conditions.

This systems-based perspective provides the foundation for the concept of bank-speed alignment, developed in the following sections. By linking athlete velocity to structural constraints, this framework provides a conceptual basis for interpreting how indoor track configuration may relate to differences in mechanical demand across events and performance environments.

## Facility design and the “track effect”

Although indoor track performances are often interpreted through athlete-centered variables, accumulating evidence suggests that facility characteristics may be associated with differences in performance conditions across venues. This phenomenon, often described informally as certain tracks being “fast” or “slow,” likely reflects a combination of structural, environmental, and competitive factors rather than a single causal mechanism.

One of the most comprehensive examinations of this effect was conducted by Barnes and Malcata (17), who analyzed more than 300,000 indoor track performances across events and facilities. Their findings demonstrated that performances varied significantly based on track configuration, with banked and oversized tracks associated with faster performances compared to flat or undersized configurations. Importantly, these differences persisted after accounting for athlete caliber and competition level, suggesting that facility design may contribute to variability in observed performances, although the relative contribution of individual factors cannot be isolated.

Anecdotally, high-performance indoor facilities such as the Boston University Track and Tennis Center (BU) have become associated with a disproportionate number of record-level performances, particularly in middle- and long-distance events. Similar patterns are observed at venues such as the Armory Track and Field Center in New York and Arena Stade Couvert in Liévin, where elite performances consistently cluster despite differences in location and competition context (Table 1). These observations are descriptive and do not establish causal relationships but highlight recurring patterns that warrant further consideration within a broader performance framework.

**Table 1:** Representative indoor track facilities illustrating variability in geometric and structural characteristics.

Facility	Location	Track system (surface & substructure)	Bank angle (°)	Curve radius	Competition context	Representative performance context
Boston University Track & Tennis Center	Boston, USA	Fixed wood-frame substructure with Mondo synthetic surface over plywood base	18°	>17.5 m	David Hemery Valentine Invitational; John Thomas Terrier Classic; elite professional indoor meets	Site of multiple world, American, and NCAA record performances; frequently associated with high-density elite middle-distance results
The Armory (Nike T&F Center)	New York, USA	Fixed wood-frame substructure with Mondo synthetic surface over plywood base	10°	<17.5 m	Millrose Games; Dr. Sander Invitational; USATF Indoor Championships	Venue for national and international competitions with numerous record-level performances
The TRACK at New Balance	Boston, USA	Hydraulic adjustable steel substructure with prefabricated Mondo synthetic surface system	0°–12°	∼17.5 m	New Balance Indoor Grand Prix; Boston College Eagle Elite Invitational; professional indoor meets	Associated with national records and top-tier collegiate and professional performances
Randal Tyson Track Center	Fayetteville, USA	Hydraulic adjustable steel substructure with prefabricated Mondo synthetic surface system	0°–12°	∼17.5 m	NCAA Division I Indoor Track & Field Championships; SEC Indoor Championships	Consistent site of NCAA championship-level and all-time performances
Reggie Lewis Center	Boston, USA	Fixed-base substructure with synthetic track surface system	8°	∼17.5 m	New Balance Indoor Nationals; MIAA State Championships; regional and national high school meets	Historically associated with competitive national-level performances
Omnisport Apeldoorn	Apeldoorn, Netherlands	Hydraulic adjustable steel substructure with prefabricated Mondo synthetic surface system	0°–12°	∼17.5 m	European Athletics Indoor Championships; Dutch National Championships	Venue for European-level championship performances
Emirates Arena	Glasgow, UK	Hydraulic adjustable steel substructure with prefabricated Mondo synthetic surface system	0°–12°	∼17.5 m	World Athletics Indoor Championships (2018); British Indoor Championships; international invitationals	Associated with international championship performances and national records
Arena Stade Couvert (Liévin)	Liévin, France	Hydraulic adjustable steel substructure with prefabricated Mondo synthetic surface system	0°–12°	∼17.5 m	Meeting Hauts-de-France Pas-de-Calais (World Athletics Indoor Tour Gold)	Frequent site of world-leading performances and world records across middle-distance events
Arena Toruń	Toruń, Poland	Hydraulic adjustable steel substructure with prefabricated Mondo synthetic surface system	0°–12°	∼17.5 m	World Athletics Indoor Championships (2021); Copernicus Cup (World Athletics Indoor Tour)	Venue for national and international record-level performances
Scandinavium	Gothenburg, Sweden	Temporary modular substructure with prefabricated synthetic track surface system	∼10°–12°	∼17.5 m	European Athletics Indoor Championships; Gothenburg Indoor Meeting (historical)	Temporary installation with championship-level competition context

Facilities were selected to illustrate variability in indoor track design characteristics, including banking configuration, substructure type, and competition context. Bank angles for hydraulic systems are presented as operational ranges reflecting typical adjustable limits, whereas fixed systems represent permanent configurations. Curve radii are reported where publicly available or inferred from standard indoor track geometry (lane-1 radius ≈ 17.5 m) and should be interpreted as representative rather than definitive engineering measurements. Temporary installations may vary across competitions. Facility characteristics were compiled from publicly available facility specifications, manufacturer descriptions, governing-body documents, and venue materials where available; however, because some facilities do not publicly report complete engineering specifications, several values should be interpreted as approximate and illustrative. Performance context descriptions are included to provide contextual examples of competition history at these venues and do not imply causal relationships between facility design and performance outcomes.

Table 1 highlights variability in indoor track design, including differences in banking configuration (fixed vs. hydraulic), bank angle (0° to ∼18°), surface composition, and underlying substructure (values are compiled from publicly available sources and are intended for illustrative purposes rather than as a comprehensive dataset). These differences are not trivial; they influence how mechanical demands are distributed between the athlete and the track surface. Facilities associated with high densities of elite performances often exhibit distinct geometric and structural characteristics, although these features cannot be interpreted in isolation from competition context. Accordingly, these observations should be interpreted as descriptive patterns rather than evidence that track geometry independently determines performance outcomes.

In addition to geometry, the structural system beneath the running surface represents an important but often overlooked component of track design. The substructure can influence surface compliance and energy return during ground contact. Although manufacturers attempt to standardize these effects, experimental work suggests that variations in surface compliance do not uniformly translate to performance differences across conditions (18). Wood-frame systems, such as those used at BU and The Armory, are more compliant and may provide elastic energy return, contributing to the “springiness” often reported by athletes. In contrast, steel or concrete substructures tend to be more rigid, potentially altering the mechanical interaction between the athlete and the surface. Although manufacturers attempt to mitigate these differences through surface engineering, variation in substructure may contribute to differences in mechanical interaction between athlete and surface, but its independent effect on performance outcomes is not directly established.

The BU track provides a particularly illustrative example. Unlike most modern indoor tracks, which typically employ hydraulically adjustable bank angles of approximately 0°–12° (Table 1), the BU track is constructed with a steeper fixed bank of approximately 18°. Mechanically, steeper banking allows a greater proportion of the ground reaction force to contribute to centripetal force production, potentially reducing the need for active lateral force generation within the simplified mechanical framework described in later sections. In addition to bank angle, the BU track incorporates a relatively large turn radius and a subtly asymmetrical banking profile, with a steeper entry into the curve and a more gradual exit. These features may facilitate smoother transitions between straight and curved sections. The track surface consists of a wood-based structural system covered with rubber-coated plywood, which may further influence the interaction between the athlete and the surface.

Taken together, these characteristics define a mechanically distinct running environment, but their specific contribution to performance outcomes cannot be isolated from other factors such as competition density, pacing conditions, and athlete selection.

A similar pattern is observed at Arena Stade Couvert in Liévin, France, which has emerged as a consistent non-U.S. venue for record-level indoor performances, particularly in middle-distance events. Although the track employs a more typical hydraulically adjustable banking system, the concentration of elite performances suggests that factors beyond bank angle alone—including competition structure, pacing conditions, and athlete selection—likely contribute to observed outcomes.

From a theoretical perspective, the influence of track geometry on performance is consistent with modeling approaches demonstrating that minimizing curvature—through increased radius or modified track design—can alter mechanical constraints during running (8). In this context, standard indoor track designs represent a compromise between performance considerations and practical constraints such as facility size and multi-sport use.

These observations indicate that performance outcomes are shaped by more than a single structural variable. Competition density, pacing strategies, athlete selection, and the broader competitive environment interact with track geometry to influence performance conditions. The atmosphere of certain facilities—including crowd proximity, noise, historical significance, and the expectation of fast times—may influence arousal, motivation, and pacing behavior (19). Research in sport psychology suggests that environments characterized by high spectator engagement and dense, high-quality fields can facilitate performance through mechanisms such as social facilitation, increased attentional focus, and optimized arousal regulation (20–22).

Facilities that consistently produce high-level outcomes may therefore function not only as mechanically distinct environments but also as contexts that amplify performance through competitive and psychological factors. The historical reputation of venues known for fast times may further influence athlete expectations and pacing decisions (21, 23, 24). In this sense, the “track effect” is best understood as an emergent outcome of interacting structural, technological, and contextual factors rather than a single underlying cause.

However, the recurring association between specific facilities and high-level outcomes indicates that structural characteristics remain a central component within this broader system. Rather than acting in isolation, these mechanical features define the conditions under which performance occurs and may interact with event-specific demands and competition context.

Importantly, performance patterns appear to differ across event types. Middle- and long-distance events show clearer clustering of record-level performances at specific venues, whereas sprint performances are more broadly distributed. These patterns are descriptive and may reflect differences in how track geometry interacts with event-specific velocity demands, rather than direct causal effects. In sprint events, higher velocities increase centripetal force requirements and may exceed the bank angles achievable in most indoor facilities, resulting in systematic under-banking within the simplified mechanical framework.

In contrast, the velocities associated with middle- and long-distance events may fall closer to the range of bank angles available in contemporary track designs. Under these conditions, mechanical demands may differ relative to sprint events, although performance outcomes remain influenced by multiple interacting factors.

Taken together, these patterns highlight the need for a framework that accounts for variability in performance conditions across indoor track environments. Rather than attributing differences solely to athlete characteristics or isolated facility features, outcomes appear to emerge from the interaction between velocity, track geometry, and contextual factors. Indoor track facilities should therefore be viewed not as equivalent competition spaces, but as environments that differentially shape performance conditions across events and competitive contexts.

## Mechanical foundations of curve running on indoor tracks

Running on a curved surface introduces mechanical demands that differ from those observed during straight-line running. Early theoretical and experimental work has demonstrated that curved running imposes distinct mechanical constraints compared to straight-line locomotion, particularly with respect to force orientation and trajectory control (25). In addition to generating propulsive forces to maintain forward velocity, athletes must also produce centripetal force to sustain a curved trajectory. The magnitude of this force is determined by both running velocity and the radius of the curve, such that faster speeds and smaller radii require greater inward-directed force.

From a physical perspective, the centripetal force (*F*) required to maintain curved motion is described by:

$$F_{centripetal}=\frac{mv^{2}}{r}$$ (1)

where *m* represents body mass, *v* is running velocity, and *r* is the radius of the curve. This relationship highlights a fundamental constraint of curved running: centripetal force requirements increase with the square of velocity, meaning that relatively small increases in speed result in disproportionately larger force demands. This relationship is consistent with prior modeling work demonstrating that curvature imposes mechanical constraints on force production and achievable velocity during curved running (8).

**Figure 1:**
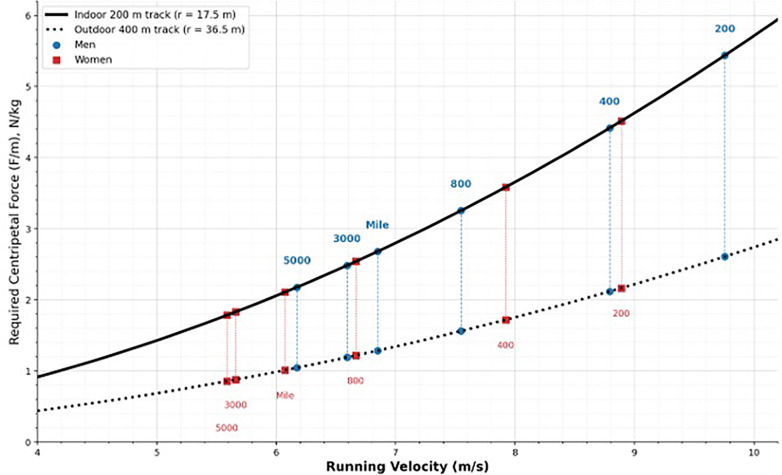
Required centripetal force as a function of running velocity for representative indoor (*r* = 17.5 m) and outdoor (*r* = 36.5 m) track geometries. Values are calculated using Equation 1 (*F* = mv^2^/*r*) assuming constant velocity and fixed radius. Event-specific markers represent approximate mean race velocities derived from elite performance benchmarks (Table 2). These values do not account for within-race velocity fluctuations, lane-dependent variation, or individual biomechanical differences and should be interpreted as illustrative rather than predictive of actual force production.

These relationships are illustrated in Figure 1, which depicts the centripetal force requirements across a range of running velocities for representative indoor (200 m) and outdoor (400 m) track geometries. Representative event velocities for men and women were derived from elite performance benchmarks (Table 2). While both relationships increase nonlinearly with velocity, reflecting the quadratic dependence described in Equation 1, the smaller radius of the indoor track results in consistently greater force demands at all speeds. Importantly, the absolute difference in required force increases as velocity increases, such that higher-speed events are associated with disproportionately greater mechanical demands. Within the scope of this analysis, these differences are interpreted as mechanical constraints rather than direct predictors of performance outcomes.

**Table 2:** Representative elite performance benchmarks and corresponding mechanical parameters across events.

Sex	Event	Performance time	Mean race velocity (m·s⁻^1^)	200 m Split (s)	Theoretical bank angle (°)^a^
Men	5,000 m	13:30	6.173	32.40	12.51
	3,000 m	7:35	6.593	30.33	14.21
	Mile	3:55	6.848	29.20	15.28
	800 m	1:46	7.547	26.50	18.36
	400 m	45.5	8.791	22.75	24.24
	200 m	20.5	9.756	20.50	29.01
Women	5,000 m	14:55	5.587	35.80	10.30
	3,000 m	8:50	5.660	35.33	10.57
	Mile	4:25	6.073	32.93	12.12
	800 m	2:00	6.667	30.00	14.51
	400 m	50.5	7.921	25.25	20.07
	200 m	22.5	8.889	22.50	24.71

Representative values reflect elite-level performance benchmarks used to approximate typical velocity ranges across events. Mean race velocity was calculated from performance time assuming constant velocity, and 200 m split times were derived accordingly. Theoretical bank angle values were calculated using Equation 4 [*θ* = tan⁻^1^(v^2^/*rg*)] assuming a constant turn radius (*r* = 17.5 m) and gravitational acceleration (*g* = 9.81 m·s⁻^2^). These calculations do not account for acceleration phases, intra-race velocity variation, lane-dependent radius differences, or individual biomechanical responses and should be interpreted as first-order approximations rather than precise or predictive performance conditions.

a Calculated under idealized conditions as described in Equation 4.

**Figure 2:**
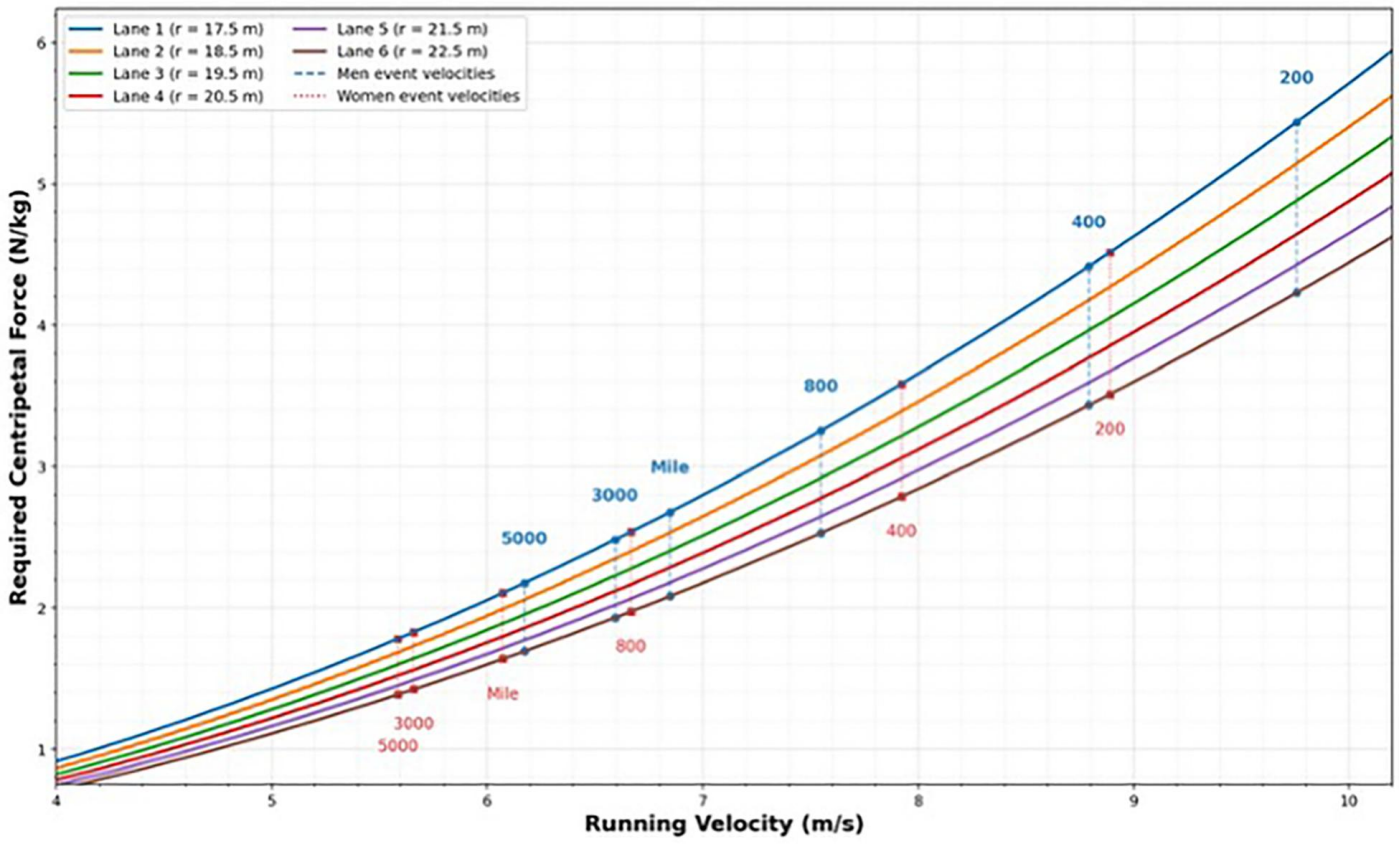
Centripetal force requirements across lane positions as a function of running velocity. Curves represent different lane radii assuming constant velocity and circular motion. Event-specific markers represent approximate velocity ranges. This analysis reflects differences in curvature and does not incorporate full biomechanical, energetic, or work–energy considerations. Accordingly, the figure should be interpreted as illustrating mechanical differences in force demand rather than direct energetic or performance consequences.

In addition to differences between indoor and outdoor track configurations, mechanical demands also vary across lane positions within a given facility. Because turn radius increases from the inside to the outside lanes, the centripetal force required to maintain curved running decreases as a function of lane position. To illustrate this effect, centripetal force requirements were calculated across lanes 1–6 of a standard indoor track, assuming a lane width of 1.0 m and a lane 1 radius of approximately 17.5 m (Figure 2).

The resulting curves demonstrate that centripetal force requirements increase nonlinearly with running velocity and are consistently highest in the innermost lanes due to their smaller radius of curvature. Although differences between lanes are modest at lower velocities, they become more pronounced as running speed increases, reflecting the velocity-squared dependence of centripetal force. These patterns indicate that mechanical demands are not uniform across lane positions, even within a single facility, and that athletes experience systematically different curvature-related constraints depending on lane assignment. These calculations assume constant velocity and fixed radii and therefore represent simplified mechanical conditions that do not account for lane-specific running lines, acceleration phases, or within-stride variability.

To generate the inward-directed forces described above, athletes adopt an inward lean, aligning the resultant ground reaction force with the center of the curve. As running velocity increases, the required lean angle also increases, placing greater demands on balance, coordination, and force production (26, 27). These adjustments alter movement mechanics relative to straight-line running and introduce asymmetrical loading patterns that must be managed throughout each step cycle (28, 29).

In addition to linear force requirements, curved running introduces a rotational component at the foot–surface interface. As the body follows a curved trajectory, the stance foot must resist outward drift while maintaining alignment with the direction of travel, increasing reliance on friction between the shoe and track surface to prevent slipping and maintain effective force application (30). Together, these demands require athletes to not only generate inward-directed force but also stabilize rotational forces that act to twist the foot relative to the surface, contributing to asymmetrical loading between limbs and increasing the mechanical complexity of force application during curved running (31).

Within this context, the mechanical properties of the running surface may further influence how forces are generated and absorbed during stance. Variations in surface stiffness have been shown to alter running mechanics and energetic cost, reflecting adjustments in how athletes interact with the ground under different structural conditions (32). From an energetic perspective, these combined demands may reflect shifts in how metabolic cost is distributed across different mechanical tasks during running. Partitioning approaches suggest that changes in force production, stabilization, and collision management can contribute to overall energetic demand, even when external performance measures remain unchanged (33).

Banked track surfaces modify these mechanical requirements by altering how forces are distributed between vertical and horizontal components. The ground reaction force generated at the foot–surface interface can be resolved into a component perpendicular to the surface (normal force) and a component parallel to the surface (friction), with the orientation of these components influenced by the bank angle. On a banked curve, a portion of the normal force generated by the surface contributes to the centripetal force requirement, reducing the need for active lateral force production by the athlete within the assumptions of the model.

The velocity at which a given combination of radius and bank angle satisfies the centripetal force requirement under simplified conditions can be expressed as:

$$v=\sqrt{rg\tan(\theta )}$$ (2)

where *θ* represents the bank angle and *g* is gravitational acceleration (9.81 m·s⁻^2^). This relationship represents a first-order mechanical reference condition derived under idealized assumptions (constant velocity, continuous motion, and negligible additional stabilizing forces), and does not represent a validated performance optimum in human running.

This relationship is inherently velocity dependent. For a given track geometry, there exists a specific velocity at which the inward component of the ground reaction force aligns with the centripetal requirement within the simplified model. On flat surfaces, this inward force must be generated entirely through friction, whereas on banked surfaces, a portion of the normal force contributes to the inward force requirement. At velocities below this condition, the inward component of the ground reaction force exceeds the centripetal demand, requiring frictional forces to act outward. At velocities above this condition, the inward component becomes insufficient, necessitating additional inward force production through friction and muscular effort to maintain the curved trajectory.

Because indoor track events span a wide range of running velocities—from sprint events through middle- and long-distance races—the mechanical conditions experienced by athletes vary across events. A bank angle that is well suited for one velocity may be mismatched for another, resulting in differences in force production, coordination demands, and mechanical constraints. These relationships describe simplified mechanical conditions and should be interpreted as illustrative approximations rather than predictive representations of performance.

## Bank-speed alignment as a unifying performance framework

The mechanical relationships described in the preceding section indicate that curved running is governed by predictable interactions among running velocity, turn radius, and bank angle (26, 27). A key implication of these relationships is that the mechanical environment of curved running is inherently velocity-dependent, such that the same track geometry may facilitate or constrain performance depending on the speed at which it is traversed.

Within this framework, a condition emerges in which the mechanical demands of curved running may differ under these conditions within the assumptions of the model. When the relationship defined by Equation 2 is satisfied, the inward component of the ground reaction force—augmented by the orientation of the running surface—aligns with the centripetal force requirement. Under these conditions, reliance on frictional compensation and active lateral stabilization may be reduced, allowing force production to be directed more efficiently toward forward propulsion within the constraints of the mechanical model (26, 29).

This relationship arises from the interaction between centripetal force requirements (as defined in Equation 1) and track geometry. On a banked surface, the orientation of the ground reaction force relative to gravity introduces a relationship between running velocity, turn radius, and bank angle. Under idealized conditions—assuming constant velocity, continuous motion, and negligible additional frictional or stabilizing forces—the bank angle at which the inward component of the ground reaction force satisfies the centripetal requirement is given by Equation 3:

$$\tan(\theta )=\frac{v^{2}}{rg}$$ (3)

Rearranging to solve for bank angle yield Equation 4:

$$\theta =\mathrm{ta}n^{-1}\left(\frac{v^{2}}{rg}\right)$$ (4)

where *θ* is the bank angle (degrees or radians), *v* is running velocity (m·s⁻^1^), *r* is the radius of curvature (*m*), and *g* is gravitational acceleration (9.81 m·s⁻^2^).

In this context, bank angle values presented in subsequent figures and tables are calculated directly from this relationship using representative velocities and radii. These calculations assume constant velocity and a fixed turn radius and therefore represent first-order mechanical approximations of curved running conditions.

This condition can be described as bank-speed alignment, referring to the relationship between running velocity, turn radius, and bank angle under which centripetal force requirements are satisfied within the assumptions of the model. Importantly, bank-speed alignment is a conceptual construct derived from simplified mechanical relationships and does not represent a validated performance optimum in human running. Rather than describing an optimal condition, it provides a mechanical reference point that can be used to interpret how track geometry may relate to velocity-dependent running conditions.

For any given track geometry, this reference condition occurs at a specific velocity, indicating that alignment is inherently velocity dependent and cannot be satisfied uniformly across events. Indoor track events span a wide range of running velocities, from sprint events to middle- and long-distance races, and therefore interact differently with a given track configuration.

**Figure 3:**
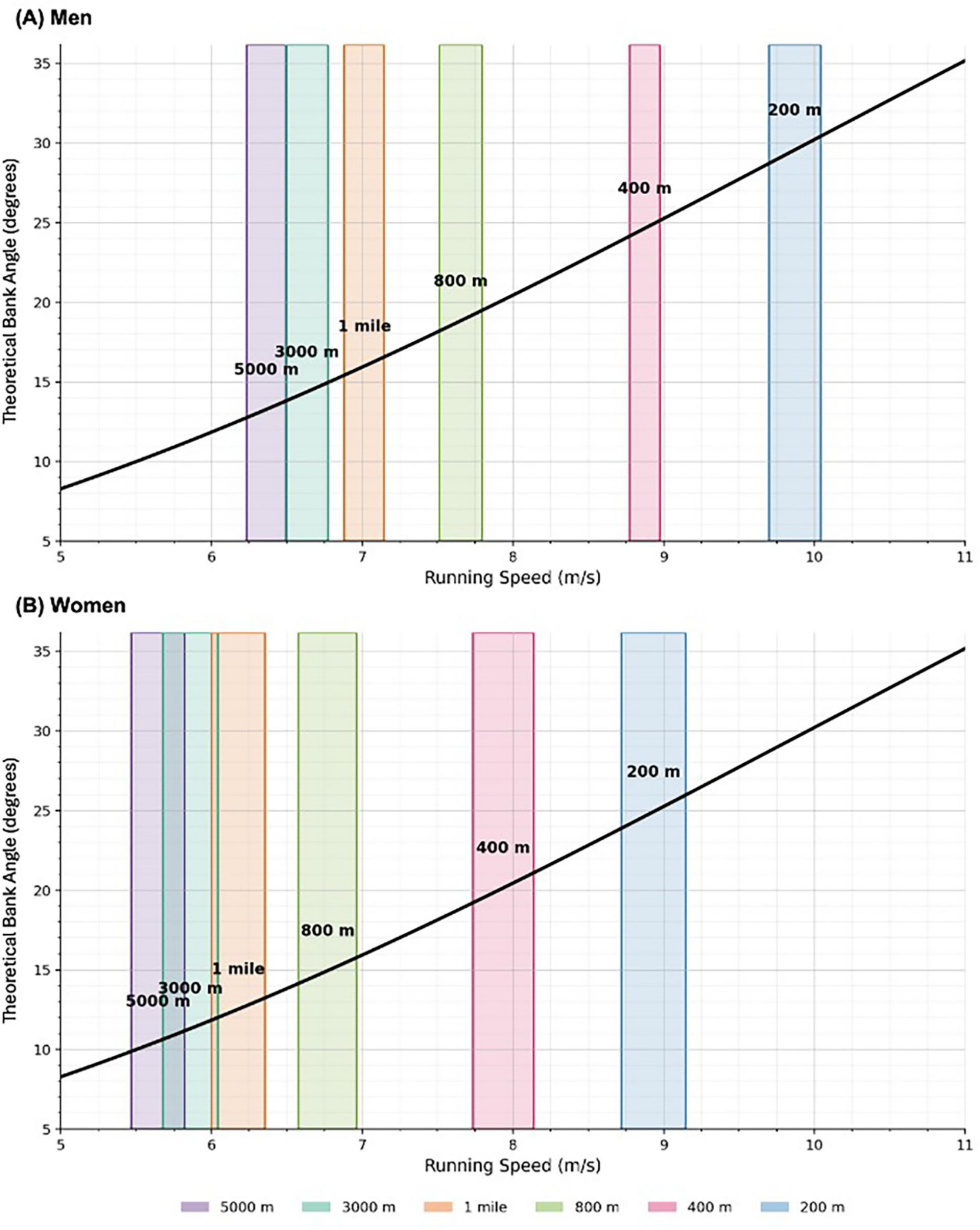
Theoretical bank angle associated with mechanical alignment as a function of running velocity for indoor track running. **(A)** Men; **(B)** women. The solid curve represents the bank angle calculated from Equation 2 under idealized assumptions, including constant velocity and a fixed turn radius of 17.5 m. Shaded vertical bands represent approximate event-specific velocity ranges derived from elite performance benchmarks (Table 3). Running velocity is based on mean race values and does not account for acceleration phases, intra-race pacing variability, or lane-dependent radius differences. Accordingly, values should be interpreted as first-order approximations intended to illustrate general velocity-dependent trends rather than precise or predictive performance conditions. The use of a single representative radius and mean velocity reflects a simplified representation of actual race dynamics.

These relationships are illustrated in Figure 3, which maps the theoretical bank angle associated with the mechanical reference condition as a function of running velocity across representative event ranges derived from performance benchmarks (Table 3). Table 3 presents illustrative values based on assumed velocities and radii and should be interpreted as conditional examples rather than event-specific predictions.

**Table 3:** Event-specific velocity and theoretical bank angle ranges derived from NCAA qualifying standards and indoor world records.

Sex	Event	NCAA Qualifying time	Velocity (m·s⁻^1^)	Theoretical bank angle (°)	World record time	Velocity (m·s⁻^1^)	Theoretical bank angle (°)
Men	5,000 m	13:22.28	6.232	12.75	12:49.60	6.497	13.81
	3,000 m	7:42.02	6.493	13.80	7:22.91	6.773	14.96
	Mile	3:53.90	6.880	15.41	3:45.14	7.148	16.57
	800 m	1:46.49	7.512	18.20	1:42.67	7.792	19.48
	400 m	45.58	8.776	24.16	44.57	8.975	25.14
	200 m	20.62	9.699	28.72	19.92	10.040	30.42
Women	5,000 m	15:14.76	5.466	9.87	14:18.86	5.822	11.17
	3,000 m	8:48.26	5.679	10.64	8:16.60	6.041	12.00
	Mile	4:28.30	5.998	11.84	4:13.31	6.353	13.23
	800 m	2:01.64	6.577	14.14	1:54.87	6.964	15.77
	400 m	51.72	7.734	19.21	49.17	8.135	21.08
	200 m	22.94	8.718	23.88	21.87	9.145	25.97

Values represent the range of elite indoor performance for each event, bounded by NCAA Division I qualifying standards (2026) and indoor world-record performances. Running velocity was calculated from official performance times assuming constant velocity. Theoretical bank angle values were calculated using Equation 2 [*θ* = tan⁻^1^(v^2^/*rg*)] assuming a constant turn radius (*r* = 17.5 m) and gravitational acceleration (*g* = 9.81 m·s⁻^2^). These calculations do not account for acceleration phases, intra-race velocity variation, lane-dependent radius differences, or individual biomechanical responses and should be interpreted as first-order approximations rather than precise or predictive performance conditions. These ranges are used to define the event-specific velocity domains illustrated in Figure 3.

**Figure 4:**
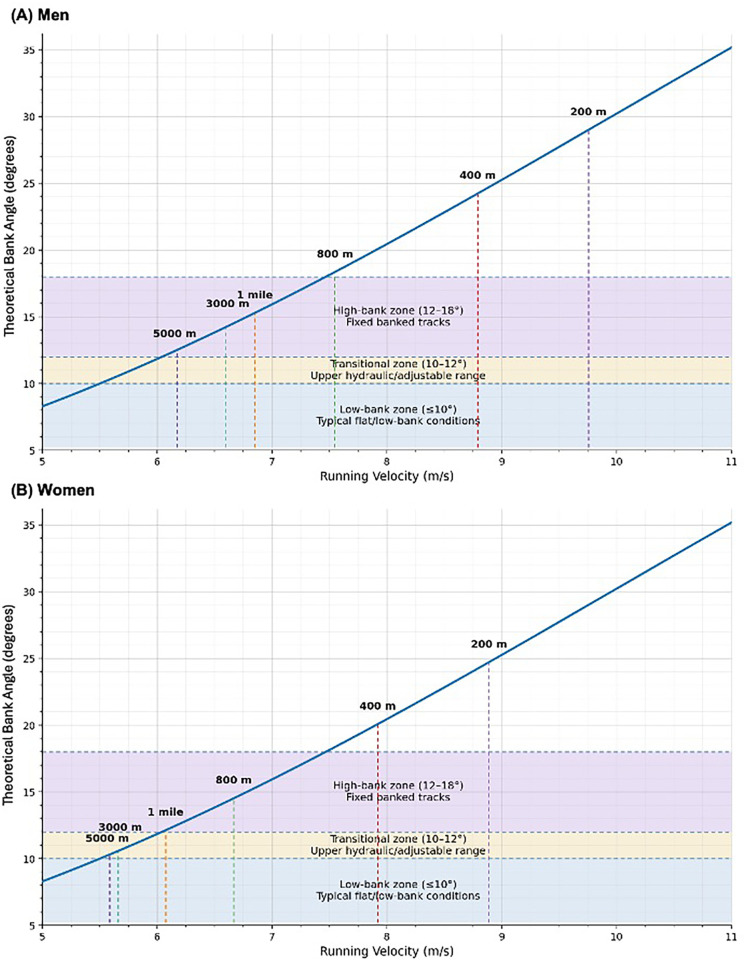
Comparison of theoretical bank angle requirements with typical indoor track design ranges. (A) Men; (B) Women. Shaded regions represent commonly observed banking configurations (e.g., low-bank ≤10°, transitional 10–12°, and high-bank 12–18°). The curve represents the theoretical relationship between velocity and bank angle derived from Equation 2 under simplified assumptions. This comparison is intended to illustrate potential differences between achievable track configurations and velocity-dependent mechanical conditions and does not imply optimal design specifications.

To place this relationship within a practical design context, typical ranges of indoor track banking can be compared with the velocity-dependent mechanical reference condition. Modern indoor track facilities generally operate within a constrained range of bank angles, reflecting trade-offs among safety, versatility, and multi-event use. Figure 4 presents this comparison by overlaying commonly observed banking ranges onto the theoretical bank angle–velocity relationship. These comparisons are intended as illustrative approximations and do not represent validated or optimal configurations for human performance.

The comparison indicates that the bank angle associated with the mechanical reference condition increases with running velocity, while the range of bank angles achievable in most indoor track facilities remains comparatively limited. As a result, different event types interact with track geometry under different mechanical conditions. Velocity ranges associated with middle- and long-distance events fall closer to commonly observed banking configurations, whereas sprint velocities extend beyond this range within the simplified model. These relationships are presented as a conceptual comparison between mechanical reference conditions and typical design constraints and do not imply optimal or prescriptive configurations.

**Figure 5:**
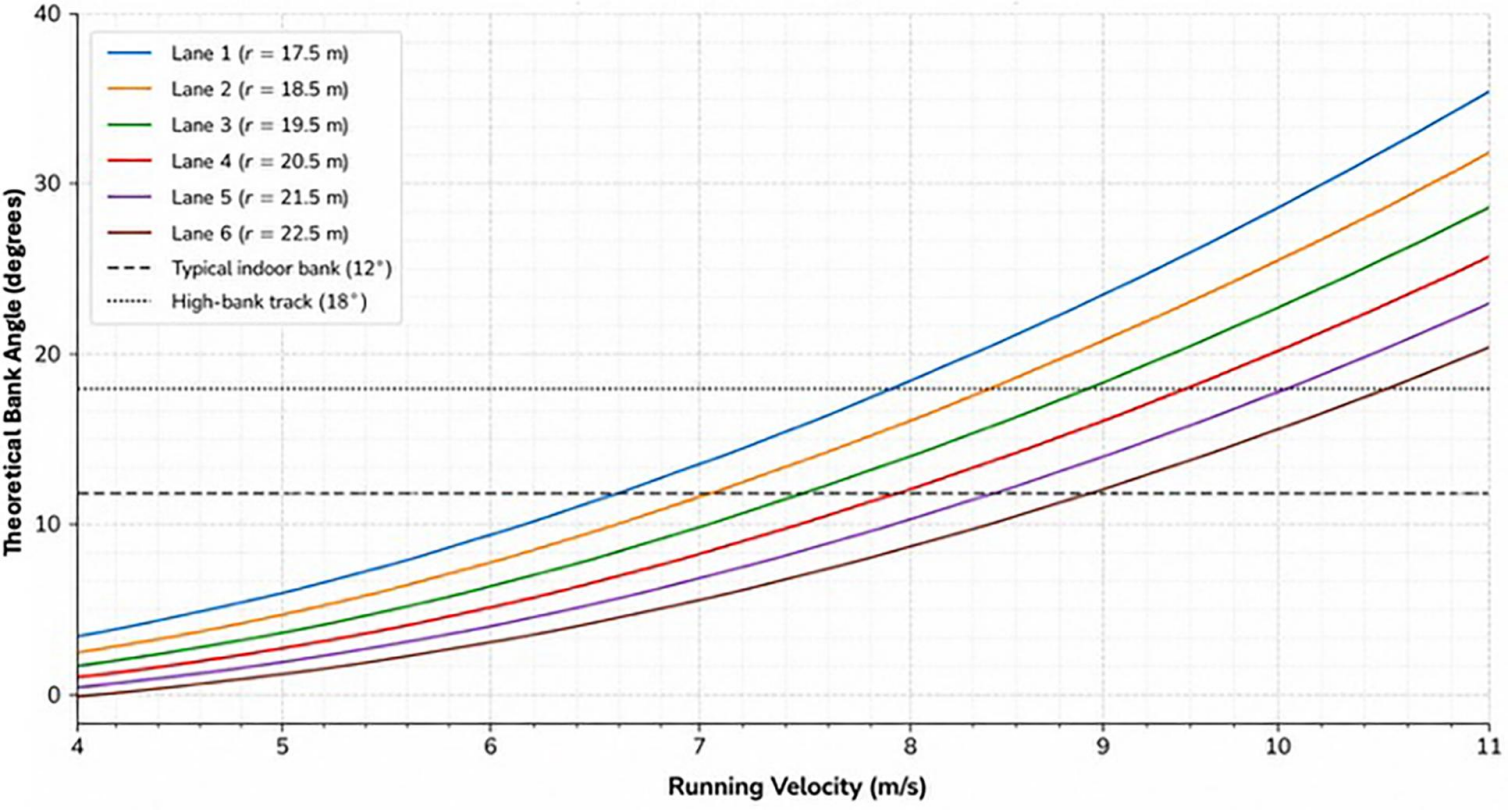
Sensitivity of the mechanical reference bank angle to running velocity and lane-specific curve radius on an indoor 200 m track. Mechanical reference bank angle (*θ*) is shown as a function of running velocity (4–11 m·s⁻^1^) for lane-specific radii corresponding to lanes 1–6 (17.5–22.5 m), assuming a constant lane width of 1.0 m. Values were calculated using a first-order banked-turn approximation $\left(\theta =\tan^{-1}\left(\frac{v^{2}}{rg}\right)\right)$, where *v* is running velocity, *r* is curve radius, and *g* is gravitational acceleration (9.81 m·s⁻^2^). Horizontal dashed lines at 12° and 18° indicate typical and upper-range bank angles observed in indoor track design. These relationships are presented as illustrative mechanical reference conditions and do not account for acceleration, pacing variability, frictional contributions, stance-phase mechanics, or athlete-specific responses.

To evaluate how sensitive this relationship is to underlying assumptions, the velocity–bank angle relationship was extended across a range of lane-specific radii corresponding to lanes 1–6 of a standard indoor track. This sensitivity analysis was included to illustrate how calculated values vary as a function of both velocity and radius, rather than to generate event-specific predictions (Figure 5).

The resulting curves demonstrate that the relationship between running velocity and the mechanical reference bank angle is nonlinear and strongly dependent on both velocity and radius. Increasing velocity produces disproportionately larger increases in the calculated bank angle, while increasing radius may systematically reduce the required angle. These results highlight that the mechanical reference condition is highly assumption-dependent and should be interpreted as an illustrative approximation rather than a precise or predictive value.

While Table 3 presents representative values based on a single assumed radius, the sensitivity analysis demonstrates that these values vary systematically when underlying assumptions are modified. This reinforces that all tabulated values should be interpreted as conditional approximations rather than fixed outputs.

When the mechanical reference condition is not met, athletes must rely more heavily on frictional forces and active stabilization to maintain curved running. These adjustments may alter how forces are generated and coordinated during movement, although the extent to which this influences performance is not directly quantified in the present framework.

Because indoor track events span a wide range of velocities, the degree to which track geometry aligns with event-specific velocity varies systematically across events. A bank angle that corresponds closely with one velocity range may be mismatched for another, resulting in different mechanical conditions across events performed on the same track.

Taken together, these relationships indicate that track geometry functions as a velocity-dependent constraint on running mechanics. Rather than providing a deterministic explanation of performance outcomes, the bank-speed alignment concept offers a conceptual framework for interpreting how mechanical conditions may vary across events and facilities.

**Figure 6:**
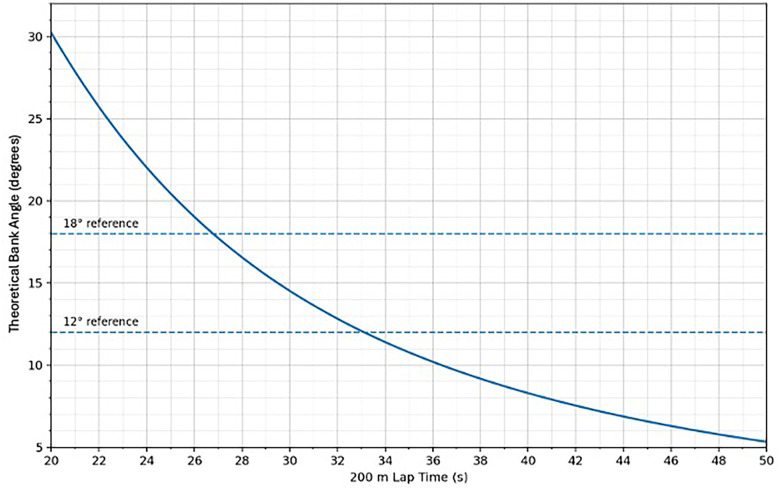
Theoretical relationship between lap time and the bank angle associated with mechanical alignment. Values are derived from the velocity-based relationship described in Equation 2 and assume constant velocity and a fixed turn radius. Reference lines at 12° and 18° represent typical indoor track banking ranges. This figure is intended to provide an intuitive representation of velocity-dependent trends and should be interpreted as illustrative rather than predictive.

This relationship can also be expressed in terms of lap time, providing an alternative representation of how the mechanical reference condition varies across performance levels. Figure 6 extends this relationship by expressing the velocity–bank angle relationship as a function of lap time.

In this representation, the bank angle associated with the mechanical reference condition decreases nonlinearly as lap time increases, reflecting the inverse relationship between velocity and centripetal force requirements. Faster performances correspond to steeper bank angles within the simplified model, whereas slower performances fall within ranges more consistent with typical indoor track configurations. Reference values at 12° and 18° provide practical benchmarks for comparison with real-world track designs.

These relationships are consistent with prior modeling approaches demonstrating that track geometry and curvature influence force production constraints and running strategy (8), while providing a simplified, velocity-dependent framework for interpreting how these effects may vary across events.

Overall, the bank-speed alignment concept is intended as a hypothesis-generating framework that links established mechanical relationships with applied performance contexts, rather than as a predictive model of performance.

## Global performance context and facility design variability

Performance outcomes in indoor track and field are often evaluated within a global context, where marks achieved across different facilities are compared directly for qualification, ranking, and record purposes. This practice implicitly assumes that performances achieved under different environmental conditions are equivalent. However, as discussed in preceding sections, indoor track facilities vary in key structural characteristics, including turn radius, bank angle, surface composition, and substructure, all of which may influence the mechanical demands of running.

From a comparative perspective, this variability introduces potential challenges when interpreting performance outcomes across facilities. Athletes competing on tracks with different geometric configurations are exposed to distinct mechanical environments, which may result in differences in force production requirements, movement coordination, and pacing strategies. Pacing strategies themselves also vary across athletes and competitive contexts, with differences observed in how runners distribute effort across laps and respond to race dynamics (19, 34). These differences are not necessarily visible in performance results alone but may influence how performances are achieved.

At the same time, performance outcomes are shaped by a range of interacting factors beyond track geometry. Performance outcomes in middle- and long-distance events also exhibit inherent variability across competitions, even under relatively controlled conditions, reflecting the combined influence of physiological, environmental, and tactical factors (35). Competition density, pacing strategies, athlete selection, and environmental conditions all contribute to performance variability. As a result, differences in performance across facilities cannot be attributed to geometry alone, and any interpretation of facility-related effects must account for these interacting influences.

Within this context, the concept of bank-speed alignment provides a framework for interpreting how structural differences between facilities may relate to performance patterns across events. Because alignment is velocity dependent, facilities with similar geometric characteristics may differentially affect events performed at different speeds. This perspective is consistent with observed patterns in which middle- and long-distance events tend to show greater clustering of high-level performances at specific venues, whereas sprint performances are more widely distributed.

Rather than implying that certain facilities inherently produce faster performances, these patterns may reflect differences in how track geometry interacts with event-specific velocity demands and competition context. For example, facilities with bank angles and radii that more closely align with the velocity ranges of middle-distance events may provide mechanical conditions that reduce compensatory demands during curved running. In contrast, sprint events, which require substantially higher velocities, may remain systematically misaligned with available track geometries regardless of facility.

These relationships highlight an important limitation of direct performance comparisons across indoor environments. While governing bodies and ranking systems treat performances as directly comparable, the mechanical conditions under which those performances occur are not standardized to the same degree as outdoor track. This does not invalidate cross-facility comparisons but suggests that such comparisons may incorporate sources of variability beyond athlete performance alone.

From a practical standpoint, these considerations have implications for how performances are interpreted in competitive and evaluative contexts. Coaches, athletes, and sport scientists often rely on performance outcomes to assess progress, make training decisions, and evaluate competitive readiness. Understanding that performance outcomes may reflect both athlete capacity and environmental context may support more nuanced interpretation of results across facilities.

This perspective is also consistent with modeling work demonstrating that track geometry influences running strategy and performance constraints, even when optimal pacing and force production are considered (8). In this sense, facility design does not determine performance outcomes but contributes to the conditions under which those outcomes are produced.

Ultimately, indoor track performance should be understood as an emergent outcome of interacting athlete, environmental, and contextual factors. Structural characteristics of the track provide a consistent set of mechanical constraints within a given facility, but these constraints vary across venues and interact with event-specific demands. The framework developed in this manuscript provides a conceptual basis for interpreting these interactions, linking differences in facility design to potential differences in mechanical demand and performance expression across events and competitive contexts.

## Mechanical implications of bank-speed misalignment

The concept of bank-speed alignment has important implications for curved running mechanics, particularly when athletes operate at velocities that do not satisfy the relationship defined by Equation 2. Under these conditions, the interaction between running velocity and track geometry becomes mechanically mismatched within the simplified model, requiring adjustments in how forces are generated and applied during curved running (26, 28, 36, 37).

From a biomechanical perspective, curved running introduces asymmetrical loading patterns relative to straight-line locomotion, driven in part by velocity-dependent centripetal force demands (28, 29, 36). Such asymmetrical loading patterns are consistent with broader observations of side-to-side differences in running mechanics, which have been linked to variations in loading distribution and movement coordination during locomotion (38). The requirement to generate centripetal force necessitates an inward lean and a redistribution of ground reaction forces, increasing mediolateral force demands (26, 36). When bank-speed alignment is approached, these forces are oriented more favorably within the simplified mechanical framework developed here, whereas under mismatched conditions, a greater proportion of force must be directed toward maintaining trajectory rather than forward propulsion within the assumptions of the model (28, 31, 37).

Banked curves also introduce vertical displacement of the running surface across lanes, particularly in events where athletes remain in fixed lanes. For example, on a track with a 12° bank angle and a lane width of approximately 1.0 m, the vertical rise between adjacent lanes is approximately 0.21 m (tan 12° ≈ 0.213), resulting in a total elevation difference of roughly 1.0–1.1 m between lane 1 and lane 6. This geometric difference indicates that athletes in different lanes experience distinct structural conditions, although the mechanical and energetic implications of this elevation difference are not directly quantified within the present framework and cannot be inferred solely from cross-sectional height differences.

However, vertical displacement represents only one component of lane-dependent mechanical variability. As shown in Figure 2, centripetal force requirements decrease systematically with increasing lane radius, such that athletes in inner lanes experience greater force demands at equivalent running velocities. Because centripetal force scales with the inverse of turn radius (*F* ∝ *v*^2^/*r*), even modest differences in lane position produce measurable differences in mechanical loading across the curve with bend sprinting mechanics and performance varying across lanes due to differences in effective radius (39).

In inner lanes, smaller radii require greater centripetal force production during ground contact, increasing the magnitude of force that must be generated within limited contact times. Because force production is constrained by the duration of ground contact at high running speeds, athletes may adjust how force is distributed across the stance phase, including changes in duty factor (27). These adjustments represent changes in force–time distribution rather than direct indicators of performance limitation within the present framework. Conversely, athletes in outer lanes experience reduced centripetal force demands due to larger radii, although this may be accompanied by differences in body positioning and movement coordination.

These mechanical demands are further influenced by how banking is distributed throughout the turn. Most modern indoor tracks employ uniform banking profiles, in which bank angle remains constant throughout the curve. While this design provides consistency and versatility, it introduces relatively abrupt transitions at the entry and exit of the turn, requiring reorientation of body position and force vectors. In contrast, asymmetrical banking profiles—such as those used in certain facilities—feature steeper entry angles and more gradual exits, which may alter how forces are applied across phases of the curve, although the effects of these designs are not directly evaluated here.

In addition to banking profile, the geometry of the track in the horizontal plane also influences how curvature is introduced. Many indoor tracks incorporate transition curves, often designed as clothoid (spiral) segments, which gradually increase curvature from the straight into the bend. These sections allow athletes to progressively adjust body lean and force application, reducing the abruptness of directional change at curve entry. However, while transition curves smooth the onset of curvature, they do not eliminate the steady-state mechanical demands imposed by the fixed radius of the turn.

Taken together, these features indicate that curved running involves a redistribution of mechanical demands across multiple domains, including force magnitude, force direction, and coordination patterns. Within the present framework, bank-speed misalignment can be interpreted as a shift in how these mechanical demands are distributed rather than as a direct determinant of performance outcomes. Under mismatched conditions, a greater proportion of force may be directed toward maintaining trajectory, which may alter how force is applied and coordinated during movement.

These mechanical considerations may have broader physiological or performance-related implications; however, such effects are not directly quantified within the present analysis and should be interpreted as areas for future investigation rather than established outcomes.

Finally, repeated exposure to asymmetrical loading patterns during curved running may alter how mechanical stress is distributed across joints and tissues. While these patterns are consistent with prior biomechanical observations, their implications for fatigue, injury risk, or long-term adaptation are not directly assessed within this framework and should be interpreted cautiously. These loading differences are consistent with broader findings indicating that asymmetrical and repetitive loading patterns can influence joint stress distribution and movement symmetry during running (40, 41).

## Applied implications for training, competition and athlete management

The following considerations are exploratory and hypothesis-generating and should not be interpreted as prescriptive recommendations derived from direct experimental evidence. The concept of bank-speed alignment provides a framework for interpreting how track geometry may relate to performance conditions, with potential implications for training, competition strategy, and athlete management.

From a training perspective, curved running introduces mechanical demands that differ from straight-line locomotion, including asymmetrical force production, altered ground reaction force orientation, and increased reliance on stabilizing musculature (26, 28). Exposure to curved running conditions may therefore be relevant to consider when preparing for indoor competition, particularly in events that involve sustained curve running. For example, sprint athletes competing in the 200 or 400 m may explore training environments that reflect the force–time constraints associated with high-speed running on small-radius turns. In addition to geometric considerations, variations in surface stiffness may also influence how athletes interact with the ground during training and competition, potentially shaping movement strategies under different conditions (32). Similarly, middle- and long-distance athletes may consider training conditions that approximate race-specific pacing and curve-running demands. However, the extent to which such exposures influence performance outcomes is not established within the present framework.

From a competition standpoint, differences in track geometry may influence how athletes experience pacing and race strategy. Because mechanical demands vary with velocity, athletes may encounter different constraints depending on both event and facility, while pacing strategies themselves vary across athletes and competitive contexts (19, 34). In events where velocities fall closer to the mechanical reference condition, athletes may be able to maintain more consistent movement patterns through the curve within the assumptions of the model. In contrast, at higher velocities, greater adjustments in force application and coordination may be required to maintain trajectory, although these effects are not directly quantified in the present analysis.

These considerations may also be relevant in championship settings, where performances are compared across rounds held within the same facility. Even within a single venue, lane assignments introduce variability in mechanical demands, with bend sprinting mechanics and performance differing across lanes due to differences in effective radius (39), suggesting that athletes may experience different curvature-related constraints depending on lane position. While such differences are partially addressed through standard competition procedures (e.g., staggered starts), their practical influence on performance execution remains unclear.

From an athlete management perspective, repeated exposure to curved running introduces asymmetrical loading patterns and stabilization demands. Monitoring how athletes respond to these conditions may be relevant in the context of training load management, particularly in indoor seasons where curve running is frequent. However, links between these mechanical conditions and fatigue, adaptation, or injury risk are not directly established within the present framework and should be interpreted cautiously.

In addition to physical considerations, athletes and coaches often make decisions about competition schedules based on perceived facility characteristics. Some venues are widely regarded as “fast,” leading athletes to preferentially compete in those environments when seeking qualifying marks or record performances. While such decisions are influenced by multiple factors—including competition quality and pacing opportunities—track geometry may represent one contributing factor within this broader decision-making context, although its independent effect cannot be isolated.

Taken together, these applied considerations highlight the importance of viewing indoor track performance within a broader systems framework. Athlete capacity, training history, competition context, and environmental structure interact to shape performance outcomes. The concept of bank-speed alignment does not prescribe specific interventions, but provides a lens through which these interactions may be interpreted.

Accordingly, practitioners should interpret these considerations cautiously, recognizing that the present framework is conceptual and that direct experimental evidence linking bank-speed alignment to specific training or performance outcomes is currently limited. Future research is required to determine how these mechanical relationships may translate into applied practice.

## Conceptual implications for track design

The concept of bank-speed alignment raises broader questions regarding how track geometry may relate to the mechanical conditions experienced across different events, and whether alternative configurations could be explored within future facility design or research contexts. These considerations are conceptual in nature and are intended to support interpretation and hypothesis generation rather than prescriptive design recommendations.

Current indoor track designs reflect a balance among performance considerations, safety, facility constraints, and multi-event functionality. Most modern tracks employ standardized dimensions and banking ranges that allow for a wide variety of events to be conducted within a single venue. As a result, track geometry is necessarily a compromise rather than a configuration tailored to any single event or velocity range.

Within the simplified mechanical framework presented in this manuscript, the relationship between velocity, radius, and bank angle suggests that no single configuration can uniformly align with the full spectrum of running velocities observed across sprint, middle-, and long-distance events. This observation does not imply that existing track designs are inadequate, but rather highlights that mechanical conditions vary across events as a function of velocity and geometry.

From a conceptual standpoint, alternative track configurations could be considered in relation to how mechanical conditions vary across velocity ranges. For example, variations in bank angle, turn radius, or transition geometry could alter how forces are distributed during curved running. Such variations may also alter how mechanical and energetic demands are distributed during curved running, although these effects are not directly quantified within the present framework (33). However, the extent to which such changes would influence performance outcomes is not established within the present framework and cannot be inferred directly from simplified mechanical relationships.

Similarly, the use of asymmetrical banking profiles or variable-radius curves has been proposed in theoretical and engineering contexts as a means of modifying curvature transitions and force application. While such designs may alter the mechanical environment of curved running, their practical feasibility, safety implications, and performance effects remain to be systematically evaluated.

It is also important to recognize that track design is constrained by factors beyond mechanical considerations. Facility size, cost, spectator visibility, and the need to accommodate multiple events impose practical limitations on how track geometry can be modified. In this context, any potential design variations must be considered within a broader framework that includes engineering feasibility, regulatory standards, and athlete safety.

From a research perspective, these considerations highlight opportunities for future investigation rather than immediate application. Experimental and modeling approaches could be used to examine how variations in track geometry influence force production, movement coordination, and pacing behavior under controlled conditions. Such work would be necessary to determine whether theoretical differences in mechanical conditions translate to meaningful differences in performance or athlete experience.

Accordingly, the present framework should be interpreted as providing a conceptual basis for exploring how track geometry may relate to running mechanics, rather than as a guide for optimizing track design. The relationships described here are derived from simplified assumptions and are intended to support hypothesis generation and further empirical study.

## Discussion

The purpose of this conceptual analysis was to examine how indoor track geometry may relate to the mechanical conditions of curved running through its interaction with running velocity. The relationships described throughout the manuscript highlight that curved running is governed by predictable interactions among velocity, radius, and bank angle, and that these interactions vary systematically across event types. Rather than providing a direct explanation of performance outcomes, this perspective offers a structured approach for interpreting how mechanical constraints may differ across indoor track environments.

A central observation emerging from this analysis is the velocity-dependent nature of the mechanical reference condition. Because indoor track events span a wide range of running speeds, a single track configuration cannot uniformly correspond to the conditions described by the simplified mechanical model. As a result, athletes competing in different events encounter distinct mechanical environments, even when performing on the same track. These differences reflect variation in how running velocity interacts with track geometry, rather than differences in athlete capability alone.

It is important to emphasize that the relationships described here are derived from simplified mechanical assumptions, including constant velocity, fixed turn radius, and idealized force application. Human running involves discrete stance phases, neuromuscular coordination, frictional contributions, and dynamic transitions during curve entry and exit. Accordingly, the mechanical reference condition should be interpreted as a first-order approximation rather than a representation of actual movement dynamics. The extent to which these simplified relationships correspond to measurable differences in performance, energetic cost, or fatigue remains to be determined through empirical investigation.

Within this context, bank-speed alignment can be viewed as a conceptual lens for interpreting variability in mechanical conditions across facilities and events. Rather than implying that track geometry determines performance outcomes, this perspective suggests that structural characteristics of the track define the constraints within which performance is produced. These constraints may interact with athlete characteristics, competition structure, pacing conditions, and environmental factors to influence how performance is expressed, although the relative contribution of each factor cannot be isolated within the present analysis.

Observations of performance clustering at specific facilities are consistent with the idea that environmental conditions vary across venues, but such patterns cannot be attributed to track geometry alone. Competition density, pacing technologies, athlete selection, and psychological factors all contribute to performance variability, consistent with broader literature demonstrating inherent variability in elite athletic performance across competitive contexts (35, 42). In this sense, indoor track performance is best understood as an emergent outcome of interacting structural, technological, and contextual influences, rather than the result of a single dominant factor.

From an applied perspective, this interpretation may support more nuanced evaluation of performance outcomes across indoor track environments. Athletes, coaches, and sport scientists often rely on performance times to assess progress and competitive readiness, frequently assuming comparability across venues. Recognizing that mechanical conditions may differ across facilities provides additional context for interpreting performance results, although the practical implications of these differences remain to be established.

These considerations also raise questions regarding the standardization of performance comparisons in indoor track and field. While governing bodies treat performances as directly comparable across facilities, structural variability introduces differences in mechanical conditions that are not explicitly accounted for. This does not invalidate current comparison systems, but suggests that performance outcomes may incorporate sources of variability beyond athlete performance alone.

Several limitations of this analysis should be acknowledged. First, the framework is based on simplified mechanical relationships and does not incorporate the full complexity of human locomotion, including muscle-level dynamics, coordination variability, and transient movement phases. Second, the use of mean velocity and fixed radii does not capture variability in pacing strategies or lane-specific running paths. Third, facility-level observations are descriptive and do not account for confounding influences such as competition density, pacing technologies, or athlete selection. Finally, the framework has not been directly validated experimentally and should therefore be interpreted as conceptual rather than predictive.

Future research may build on this work by integrating more detailed biomechanical and physiological models of curved running, as well as by examining how athletes respond to different track configurations across events. Experimental studies measuring ground reaction forces, movement patterns, and metabolic cost during curved running would provide valuable insight into the practical implications of these relationships. In addition, large-scale performance datasets may help further clarify how facility characteristics interact with competition structure and athlete characteristics to shape observed outcomes.

In conclusion, indoor track performance reflects the interaction between athlete characteristics, environmental constraints, and competition context. The concept of bank-speed alignment provides a conceptual basis for interpreting how track geometry may relate to the mechanical conditions of running across events and facilities. This perspective is intended as an interpretive and hypothesis-generating approach rather than a predictive model, and its primary contribution lies in guiding future empirical investigation rather than providing definitive explanations of performance outcomes.
